# Challenges in Diagnosis and Functional Assessment of Coronary Artery Disease in Patients With Severe Aortic Stenosis

**DOI:** 10.3389/fcvm.2022.849032

**Published:** 2022-03-11

**Authors:** Srdjan Aleksandric, Marko Banovic, Branko Beleslin

**Affiliations:** ^1^Cardiology Clinic, University Clinical Center of Serbia, Belgrade, Serbia; ^2^Faculty of Medicine, University of Belgrade, Belgrade, Serbia

**Keywords:** aortic stenosis, coronary artery disease, myocardial ischemia, transcatheter aortic valve replacement, fractional flow reserve, instantaneous wave-free ratio

## Abstract

More than half of patients with severe aortic stenosis (AS) over 70 years old have coronary artery disease (CAD). Exertional angina is often present in AS-patients, even in the absence of significant CAD, as a result of oxygen supply/demand mismatch and exercise-induced myocardial ischemia. Moreover, persistent myocardial ischemia leads to extensive myocardial fibrosis and subsequent coronary microvascular dysfunction (CMD) which is defined as reduced coronary vasodilatory capacity below ischemic threshold. Therefore, angina, as well as noninvasive stress tests, have a low specificity and positive predictive value (PPV) for the assessment of epicardial coronary stenosis severity in AS-patients. Moreover, in symptomatic patients with severe AS exercise testing is even contraindicated. Given the limitations of noninvasive stress tests, coronary angiography remains the standard examination for determining the presence and severity of CAD in AS-patients, although angiography alone has poor accuracy in the evaluation of its functional severity. To overcome this limitation, the well-established invasive indices for the assessment of coronary stenosis severity, such as fractional flow reserve (FFR) and instantaneous wave-free ratio (iFR), are now in focus, especially in the contemporary era with the rapid increment of transcatheter aortic valve replacement (TAVR) for the treatment of AS-patients. TAVR induces an immediate decrease in hyperemic microcirculatory resistance and a concomitant increase in hyperemic flow velocity, whereas resting coronary hemodynamics remain unaltered. These findings suggest that FFR may underestimate coronary stenosis severity in AS-patients, whereas iFR as the non-hyperemic index is independent of the AS severity. However, because resting coronary hemodynamics do not improve immediately after TAVR, the coronary vasodilatory capacity in AS-patients treated by TAVR remain impaired, and thus the iFR may overestimate coronary stenosis severity in these patients. The optimal method for evaluating myocardial ischemia in patients with AS and co-existing CAD has not yet been fully established, and this important issue is under further investigation. This review is focused on challenges, limitations, and future perspectives in the functional assessment of coronary stenosis severity in these patients, bearing in mind the complexity of coronary physiology in the presence of this valvular heart disease.

## Introduction

Degenerative aortic stenosis (AS) is the most common valvular heart disease in both western and developed countries, affecting mainly individuals older than 60 years (1–4). Co-existing coronary artery disease (CAD) is present in more than 50% of patients with severe AS over 70 years of age and in more than 65% of patients with severe AS over 80 years of age (2, 3, 5). Both conditions are strongly associated with age and risk factors for degenerative AS are similar to those seen in atherosclerosis including male sex, smoking, hypertension, diabetes, low-density lipoprotein (LDL) cholesterol, and C-reactive protein (2, 4–6). According to current guidelines of the European Society of Cardiology (ESC) and of the American College of Cardiology/American Heart Association (ACC/AHA), coronary artery bypass grafting (CABG) is recommended (class I, level of evidence C) in addition to surgical aortic valve replacement (SAVR) during the same surgical procedure in patients with a severe symptomatic AS and concomitant coronary stenosis ≥70% diameter stenosis (DS) or ≥50% DS in case of left main (LM) stenosis, whereas CABG should be considered in AS-patients with concomitant stenosis ≥50–70% DS in non-LM coronary arteries (class IIa, level of evidence C) (7–9). Two large studies demonstrated that significant CAD which was not revascularized at the time of SAVR was associated with increased risk of adverse short- and long-term outcomes (10–12). In contrast, several surgical nonrandomized studies identified concomitant CABG as an independent predictor of short and long-term mortality among patients undergoing SAVR, especially in elderly patients above 80 years old (5, 10, 13–19). Accordingly, over the last decade, transcatheter aortic valve replacement (TAVR) has been established as the treatment of choice for patients with severe symptomatic AS who are deemed inoperable or at high-risk for SAVR. These high-risk AS-patients are frequently elderly with different comorbidities beyond CAD, such as chronic kidney disease, diabetes, hypertension, or impaired left ventricle (LV) systolic and/or diastolic function (5). Thus, the prevalence of CAD is extremely high in this patient population, ranging up to 75% (5). In recent years, published large randomized trials and meta-analyses demonstrated non-inferiority and even superiority concerning major adverse cardiac events (MACE) favoring TAVR over SAVR across the spectrum of AS-patients, irrespective of baseline surgical risk (20–24). In addition, recently presented the Aortic Valve ReplAcemenT vs. Conservative Treatment in Asymptomatic SeveRe Aortic Stenosis (AVATAR) randomized trial demonstrated benefit of early SAVR in asymptomatic patients with severe AS and normal LV ejection fraction (25). Expert consensus opinion (class IIa, level of evidence C) highlights that percutaneous coronary intervention (PCI) should be considered in AS-patients with a primary indication to undergo TAVR who have stenosis of at least 70% DS in the proximal segments of epicardial coronary arteries that subtend a large area of myocardium at risk (7, 10). This has been recently challenged with the results from the Assessing the Effects of Stenting in Significant Coronary Artery Disease Prior to Transcatheter Aortic Valve Implantation (ACTIVATION) trial (26). Obviously, the optimal management of AS-patients with concomitant CAD in patients undergoing TAVR remains controversial due to the heterogeneity of available data, and the clinical relevance of PCI performed before or immediately after TAVR remains to be determined (2, 5, 27–29). Yet, the purpose of this review is to focus on challenges, limitations, and future perspectives in the functional assessment of coronary artery stenosis in patients with AS, bearing in mind the complexity of coronary physiology in the presence of this valvular heart disease.

## Coronary Artery Disease Assessment in Patients With Aortic Stenosis

Exertional angina is the most common presenting symptom in patients with obstructive CAD (2, 4, 6). However, angina is also often present in patients with severe AS, even in the absence of obstructive CAD, as LV oxygen demand exceeds supply (2, 4, 6). The presence of AS increases LV afterload and wall stress resulting in concentric LV hypertrophy as a compensatory mechanism to normalize LV wall stress and maintain LV systolic function (30). Consequently, cardiac output in most of these patients is preserved for many years despite an elevated LV afterload. Hence, the LV oxygen demand is increased by LV afterload, LV hypertrophy, inotropic state and prolonged systolic ejection phase, particularly in the LV subendocardium (2, 4, 6, 30). In such condition, resting coronary blood flow is sustained due to vasodilation of intramyocardial arterioles induced by autoregulation phenomenon. However, coronary blood flow is a significantly diminished during exercise or tachycardia which is usually documented by reduced coronary flow reserve (CFR) during adenosine-induced maximal hyperemia (2, 4, 6, 30). There are 3 possible mechanisms for impaired CFR during exercise or tachycardia in AS-patients with LV hypertrophy: (1) reduced diastolic filling time with subsequent low coronary perfusion pressure; (2) elevated LV diastolic filling pressure with subsequent compression of the LV endocardium and subendocardial hypoperfusion; and (3) arteriolar remodeling, perivascular fibrosis and relative decline in myocardial capillary density as a consequence of prolonged LV hypertrophy (2, 4, 6, 30–33). These mechanisms are responsible for the diminished LV oxygen supply in these patients, especially during exercise or tachycardia (2, 4, 6, 30–33). As a result of concomitant increased LV oxygen demand and diminished LV oxygen supply, exercise-induced myocardial ischemia and exertional angina may occur. Moreover, persistent myocardial ischemia leads to extensive myocardial fibrosis and subsequent coronary microvascular dysfunction (CMD) which is defined as reduced coronary vasodilatory capacity below ischemic threshold (2, 4, 6). Therefore, angina, as well as non-invasive stress tests, have a low specificity and positive predictive value (PPV) for the assessment of epicardial coronary stenosis severity in AS-patients (2, 4, 6). A case in point, approximately 20% of patients with severe AS and non-ischemic exercise-testing have significant CAD at subsequent coronary angiography defined as the visually assessed coronary stenosis >70% DS or 50–70% DS with fractional flow reserve (FFR) ≤ 0.80 (34).

In patients with severe AS, the conventional exercise-stress test and thallium-201 exercise-scintigraphy are generally found to be inaccurate with low specificity for CAD assessment since clinical symptoms and baseline ECG abnormalities are neither specific nor sensitive (35–41). Exercise-induced myocardial ischemia may occur in these patients, even in the absence of CAD (35–41). While exercise-stress testing is contraindicated for symptomatic patients with severe AS, it has clinical relevance for identifying those asymptomatic AS-patients who are at high-risk of poor prognosis (8, 9, 35–41). Pharmacological stress tests such as single photon emission computed tomography (SPECT), positron emission tomography (PET) or cardiac magnetic resonance (CMR) with different vasodilators (adenosine, dipyridamole or regadenoson) have been shown to be a valuable alternative to exercise-stress testing for the CAD assessment in these patients (35–41). These pharmacological tests were found to be safer and more accurate for identifying functionally significant CAD compared to exercise-stress testing in AS-patients (35–41). However, due to its limited specificity and PPV, a more sophisticated diagnostic tools were developed for assessing the coronary stenosis severity in these patients. Given the limitations of noninvasive stress tests, invasive coronary angiography remains the standard examination for determining the presence and severity of CAD in AS-patients, although angiography alone has poor accuracy in the evaluation of its functional severity (5, 6, 28). Current ESC and ACC/AHA guidelines for the management of patients with valvular heart disease recommend coronary angiography before aortic valve replacement (AVR) in symptomatic men and premenopausal women with at least one CAD risk factor >35 years old, in all asymptomatic men >45 years old and in all women >55 years old (8, 9).

Several studies suggest that coronary computed tomography angiography (CCTA) is a reasonable alternative to invasive coronary angiography for assessing CAD before AVR in AS-patients who have a low probability of CAD or in whom invasive coronary angiography is technically not feasible or associated with a high-risk (8–12, 19, 42–57). Compared with invasive coronary angiography, it has been shown that electrocardiogram-gated CCTA has high sensitivity and negative predictive value (Sn: 89–100%; NPV: 91–100%), but low specificity and positive predictive value (Sp: 37–99%; PPV: 8–85%) in detecting angiographically significant coronary stenosis defined as >50% DS (8–12, 19, 42–57). Consequently, CTCA may be useful in excluding angiographically significant stenosis among patients at a low-risk of atherosclerosis (8–12, 19, 42–57). Chieffo et al. demonstrated that using CCTA for CAD screening prior to TAVR reduces the need for invasive coronary angiography by approximately 80%, potentially lowering both overall cost and length of hospitalization without increasing the risk of ischemic cardiovascular events (57). Invasive coronary angiography may therefore be performed when CCTA is contraindicated, fails to assess coronary anatomy, or reveals an angiographically significant proximal coronary artery lesion (8–12, 19, 42–58). However, CCTA alone cannot assess the functional significance of coronary stenosis because it provides only anatomical information about the presence and extent of CAD, and can overestimate stenosis severity, especially in the presence of high calcium scores (59–64). Recently, FFR derived from CCTA (FFR_ct_) has developed as the novel noninvasive method that provides both anatomical and functional evaluation of CAD (59–64). It has been shown that FFR_ct_ has high sensitivity and negative predictive value (Sn: 74–88%; NPV: 90–92%), but low specificity and positive predictive value (Sp: 60–82%; PPV: 41–74%) to identify functionally significant coronary stenosis defined as FFR ≤ 0.80 (59–64). In the Computed Tomography-Derived Fractional Flow Reserve in Patients with Severe Aortic Stenosis (CAST-FFR) study similar diagnostic accuracy were observed among patients with severe AS and co-existing CAD in pre-TAVR settings (65). These findings suggest that noninvasively measured FFR_ct_ before TAVR may accurately and safely exclude ischemia-driven coronary lesion and reduce the need for invasive coronary angiography in AS-patients (57–65). Further studies are required to determine the clinical utility of FFRct regarding pre-TAVR diagnostic accuracy and outcomes at longer follow-up in these patients.

## Pathophysiological Mechanisms of Exertional Angina in the Presence of Aortic Stenosis

Developing exertional angina dramatically worsens the prognosis of patients with AS, which is why AVR is highly indicated in these patients (8, 9, 66–69). Several studies demonstrated that impaired myocardial and/or coronary flow reserve, defined as the maximal hyperemic to resting myocardial or coronary blood flow ratio, is a key mechanism for developing exertional angina in patients with severe AS without obstructive CAD (67–77). Importantly, coronary microvascular function is additionally impaired is patients who have AS and concomitant diabetes, which is a common finding (78, 79). Using adenosine-stress cardiac magnetic resonance (CMR) imaging, Ahn et al. showed that the semiquantitative myocardial perfusion reserve index (MPRI) was significantly lower in the group of symptomatic patients with severe AS and angiographically normal coronary arteries compared to the group of asymptomatic AS-patients, as well as compared to the normal control group (67). Similar findings were observed in other studies evaluating both noninvasive and invasive myocardial and/or coronary flow reserve during adenosine-induced maximal hyperemia (68–77). Likewise, microcirculatory resistance in these patients was significantly higher during maximal hyperemia but significantly lower under baseline conditions in comparison to the control group (68–77). Of note, several previously published studies also noted that myocardial and/or coronary blood flow during hyperemia was markedly reduced in AS-patients who also had LV hypertrophy in comparison to normal subjects, but not at rest (67–77, 80). These findings implicate that the vasodilatory capacity of intramyocardial arterioles in severe AS-patients with LV hypertrophy but without CAD is already exhausted by the autoregulation phenomenon to maintain resting coronary blood flow in response to increased LV mass and LV oxygen demand, and therefore, the vasodilatory effect of adenosine on microcirculatory resistance is limited during maximal hyperemia (67–77, 80). Hence, a significantly reduced coronary vasodilatory capacity during exercise or stress testing may occur in severe AS-patients even in the absence of CMD, as a result of the increased resting perfusion associated with LV hypertrophy and high-pressure LV overload rather than reduced perfusion during testing (81).

Furthermore, Steadman et al. using adenosine-stress CMR imaging with late-gadolinium enhancement (LGE) identified LV hypertrophy and myocardial fibrosis, rather than the AS severity, as the major determinants of impaired myocardial perfusion reserve (MPR) in symptomatic patients with severe AS (80). Similar findings were observed in the study by Zhou et al. (82). Additionally, MPR was the only independent predictor of reduced aerobic exercise capacity (peak VO2) during cardiopulmonary exercise testing in these patients, whereas echocardiographic and CMR measures of the AS severity were not (80). It is well known that the development of LV hypertrophy in AS-patients is an adaptive and compensatory physiological response to reduce LV wall stress and preserve cardiac output (76, 80). However, LV remodeling is associated with arteriolar remodeling, perivascular fibrosis, and capillary rarefaction, which result in the development of myocardial fibrosis and subsequent CMD at a later stage of the disease (67, 76, 80, 83). Contrary to the previous study, Rajappan et al. found that both hyperemic diastolic filling time and AS severity were more important determinants of MPR in severe AS-patients without CAD compared to LV hypertrophy (76). This study revealed that reduced hyperemic diastolic filling time during stress testing in these patients, which was directly associated with the AS severity quantified as the aortic valve area <1.0 cm^2^, leads to impaired myocardial blood flow in LV subendocardium and subsequent decrease in the subendocardial-to-subepicardial perfusion ratio (76). This inconsistency with previously mentioned studies might be due to a small number of AS-patients (only 20) included in the Rajappan study who were predominantly asymptomatic. Therefore, in patients with severe AS and normal coronary arteries, the impaired subendocardial-to-subepicardial perfusion ratio with an absolute reduction in subendocardial perfusion below the ischemic threshold may occur during exercise or stress testing which simultaneously reduces diastolic filling time and increases LV pressures due to tachycardia and increased LV oxygen demand (81, 82). Additionally, it has been noted that only 60% of symptomatic patients with isolated AS had CMR-quantified MPRI values below the ischemic threshold, and vice-versa, that 60% of those who were asymptomatic had MPRI values above the same threshold (67, 81). Based on these findings, the impaired myocardial and/or coronary flow reserve as the primary cause of myocardial ischemia and exertional angina in AS-patients without CAD may occur not only due to the presence of CMD but also due to different hemodynamic conditions that mainly affects diastolic filling time and diastolic perfusion at subendocardial level (81). Therefore, CMD is not equivalent to a reduced myocardial and/or coronary flow reserve in these patients and is characterized by reduced myocardial perfusion throughout the LV wall (transmural myocardial perfusion) without a subendocardial-to-subepicardial perfusion gradient (81). Invasive measurements of microcirculatory resistance index (IMR) by thermodilution technique (with dual-sensor [pressure and temperature] wire) or hyperemic microcirculatory resistance (HMR) with dual-sensor (Doppler and pressure) wire are the methods of choice for the assessment of CMD (81, 84–88). However, IMR and HMR cannot distinguish the presence of CMD from other hemodynamic disorders that result in subendocardial hypoperfusion and ischemia. For this reason, several authors suggest CMR or PET imaging in AS-patients as a noninvasive diagnostic tool for measuring separately absolute subepicardial and subendocardial perfusion and their ratio (81, 84, 89–94).

Moreover, MPR assessed by PET imaging has been found to further decrease with worsening degrees of LV remodeling and the occurrence of systolic LV dysfunction (82). The study also showed that the annual incidence of major adverse cardiac events (MACE) including death, nonfatal myocardial infarction, hospitalization for heart failure or AVR was significantly higher in the group of AS-patients with impaired MPR regardless of their systolic LV functional status, whereas among patients with normal systolic LV function, those with impaired MPR had a significantly higher rate of MACE (82). In the PRognostic Importance of MIcrovascular Dysfunction in Aortic Stenosis (PRIMID-AS) Study, reduced MPR was found to be an independent predictor of symptom onset in initially asymptomatic patients with moderate-to-severe AS (41). The echocardiographically derived impaired coronary flow reserve (CFR) was also shown to be an independent predictor of adverse outcomes in patients with severe AS and nonobstructive epicardial coronary stenosis (95). The above cited studies suggest that reduced coronary vasodilatory capacity should be used not only as a risk-marker, but also could be used as an early sign of pathologic LV remodeling in AS-patients which indicates subendocardial ischemia at an early stage of the disease (82). Previous findings indicate that subendocardial ischemia is a result of synergistic interaction between increased intracavitary LV pressure and systolic extravascular compression, reduced diastolic filling time, low coronary perfusion pressure, and impaired coronary vasodilatory capacity (82). Likewise, the presence of CMD as a consequence of extensive myocardial fibrosis at an advanced stage of AS is considered to be a key indicator for maladaptive LV remodeling (67, 76, 77, 80, 83). These potential mechanisms underlying myocardial ischemia at early and late stages of AS require clarification in further prospective randomized trials.

## Coronary Hemodynamics After Transcatheter Aortic Valve Replacement (TAVR)

Wiegerinck et al. demonstrated that TAVR induces an immediate increase in hyperemic coronary flow velocity and a concomitant decrease in HMR, resulting in an immediate increase in CFR (77). However, CFR was significantly lower following TAVR compared to normal subjects despite high acute procedural success in this study, mainly due to unchanged resting hemodynamics before and after TAVR (77). Two studies regarding coronary wave intensity analysis also found that CFR did not improve immediately after TAVR due to unaltered resting coronary flow velocities (96, 97). Contrary, in the study by Stoller et al., a thermodilution-derived CFR and IMR were not improved immediately after TAVR regardless of the presence of angiographically significant fixed coronary stenosis (>50% DS) (98). These studies showed that changes of both CFR and microcirculatory resistance (HMR or IMR) are not associated with the acute reduction of LV afterload and outflow gradient in patients with severe AS following TAVR (77, 98, 99). It seems that autoregulatory microvascular tone in response to LV hypertrophy remains unaffected immediately after TAVR, despite an immediate decrease in both LV afterload and extravascular compression of the intramyocardial arterioles (77, 98). Hence, it is believed that complete restoration of coronary vasodilatory capacity could be improved only several months or even years after TAVR following the regression of LV hypertrophy (70, 77, 98). This was supported by several studies revealing that significant improvement in CFR was achieved only with longer-term follow-up after TAVR following LV hypertrophy regression (70, 99–103). In two of these studies, significant improvement in CFR at 6 and 30 months after AVR was mainly attributable to an increase in coronary blood flow during maximal hyperemia, whereas resting coronary flow remained similar before and after TAVR or SAVR, respectively (100, 102). The small prospective study by Vendrik et al. regarding the longer-term effects of TAVR on invasively measured coronary hemodynamics by dual-sensor (Doppler and pressure) wire revealed an ongoing increase in hyperemic coronary flow as well as in CFR immediately after TAVR and 6 months after the procedure compared with pre-TAVR values (103). Hyperemic microcirculatory resistance was simultaneously continued to decrease in a similar manner as the CFR increased during 6 months follow-up (103). On the other hand, resting coronary flow as well as resting microcirculatory resistance remain unchanged (103). Contrary, in the other two studies, significant improvement in CFR at 12 months after AVR was closely related to decreasing in resting coronary blood flow after SAVR, whereas hyperemic coronary flow remained equivalent (70, 99). Rajappan et al. also demonstrated that a decrease in resting coronary blood flow and subsequent improvement in CFR 12 months after SAVR was primarily associated with the reduction in extravascular compression and concomitant prolongation of hyperemic diastolic filling time with improvement in diastolic myocardial perfusion rather than the regression of LV hypertrophy (99). This hypothesis is supported by the findings that patients with AS experience relief of anginal symptoms immediately after AVR, even before LV hypertrophy regression has occurred (99). Accordingly, acute anginal symptoms relief immediately after AVR could not be explained by the CFR changes alone due to its limited improvement. Rajappan et al. also noted that CFR changes before and after TAVR significantly correlate with changes in hyperemic diastolic filling time, rather than with changes in LV mass (76, 99). They concluded that the diastolic filling time is an important determinant of CFR and its interaction with AS severity might contribute to the development of myocardial ischemia and/or angina in AS-patients (76, 99). This hypothesis is supported in previously published study by Ferro et al. who found that the diastolic filling time at ischemic threshold during exercise- or pacing-induced tachycardia may indirectly predict the functional significance of coronary stenosis in patients without AS (104). Authors noted that the occurrence of myocardial ischemia and/or angina in these patients is mostly determined by the interaction between reduced diastolic filling time and coronary stenosis severity (104). Considering all these findings, it appears that anginal symptoms relief immediately after AVR is a result of acute diminish in LV oxygen demand driven by decrease in LV afterload, shortened systolic ejection phase and extravascular decompression of microcirculation, and concomitant acute increase in LV oxygen supply driven by mechanical relief of aortic valve obstruction, prolonged diastolic filling time and limited improvement in coronary blood flow and myocardial perfusion, particularly during exercise or tachycardia (30, 76, 77, 96–98). Contrary, while the LV hypertrophy persists following AVR, its regression may continue for months or years thereafter leading to a progressive restoration of coronary vasodilation capacity and CFR (77, 81, 99–103). However, in those AS-patients with extensive myocardial fibrosis, coronary vasodilatory capacity may not be resolved despite LV hypertrophy regression. Having in mind that diastolic filling time is a major determinant of myocardial ischemia and/or angina in AS-patients, it would be of great importance to develop a test that could identify its critically short duration that causes ischemia (30). The predefined critically short diastolic perfusion time could be potentially used as a guide for timing AVR and further studies with AS-patients should be conducted to examine this hypothesis.

In coronary wave intensity analysis studies, the prompt symptoms relief after TAVR could be explained by two concomitant pathophysiological mechanisms. First, early- and mid-systolic forward compression wave (the dominant forward-traveling pushing wave) is depressed and delayed in the presence of AS and promptly regained after TAVR (97). Ahmad et al. found that systolic coronary blood flow is reduced at rest and during hyperemia due to concomitant obstruction of LV ventricular emptying by the stenotic aortic valve, decrease aortic flow and high extravascular compression of microcirculation caused by an increase in LV afterload (105–107). After TAVR, systolic coronary blood flow is improved at rest and during hyperemia as a result of better LV ventricular emptying through the repaired aortic valve, increase aortic flow and extravascular decompression of microcirculation due to lower LV afterload (105, 108). Second, the magnitude of early-diastolic suction wave (the dominant backward-traveling suction wave) at rest was found to be significantly higher in AS-patients with accompanied LV hypertrophy compared with normal subjects, and it increased further with increasing AS severity (96, 97). The higher magnitude of resting early-diastolic suction wave in AS-patients is mainly related to improved propagation of this wave caused by microcirculatory vasodilation in response to LV hypertrophy and high LV oxygen demand (96, 97). During tachycardia in the presence of severe AS, this wave paradoxically decreases due to the decoupling of regulatory mechanisms for maintenance of normal coronary blood flow and myocardial perfusion (96). Immediately after TAVR, the early-diastolic suction wave decreases at rest, and increases during tachycardia, as a result of an abrupt decrease in LV afterload, extravascular decompression of microcirculation and recoupling of regulatory mechanisms of normal myocardial perfusion (96). However, despite significant improvement of the early-diastolic suction wave at rest and during hyperemia, CFR immediately after TAVR remains unaltered or slightly increased primarily due to high LV end-diastolic pressure associated with LV hypertrophy affecting diastolic myocardial perfusion (77, 96). Accordingly, it seems that the improvement of systolic coronary blood flow has a dominant role in instant angina symptom relief after TAVR as a result of decreased systolic subendocardial compression due to lower LV afterload and perfusion redistribution from nonischemic to subendocardial ischemic areas, which consequently improved subendocardial ischemia (97, 105).

## The Assessment of Coronary Stenosis Severity in As Patients

To overcome limitations of noninvasive tests and coronary angiography regarding the functional assessment of coronary stenosis severity in AS-patients, the well-established invasive physiological indices, such as FFR and instantaneous wave-free ratio (iFR), are now in focus, especially in the contemporary era with a rapid increment of TAVR. Based on large clinical trials, current guidelines recommend both hyperemic and nonhyperemic indices as a reference invasive physiologic measurement for the assessment of coronary stenosis severity (7, 109, 110). The use of these physiological indices to guide coronary revascularization in patients with CAD improves clinical outcomes compared with treatment based on angiography alone (111–115). However, the optimal method for evaluating myocardial ischemia in patients with AS and co-existing CAD has not yet been fully established, and this important issue is under further investigation. Studies evaluating the use of FFR and iFR in patients with severe AS and co-existing CAD before and after TAVR are summarized in Table 1.

**Table 1:** Studies evaluating the use of fractional flow reserve (FFR) and instantaneous wave-free ratio (iFR) in patients with severe aortic stenosis (AS) and co-existing coronary artery disease (CAD) before and after transcatheter aortic valve replacement (TAVR).

**Authors** **(Ref #)**	**Citation**	**Study design**	**Number of patients and coronary lesions**	**Conclusion**
Wiegerinck et al. (77)	Circ. Cardiovasc. Interv. 2015.	Prospective, observational study: intracoronary pressure and flow velocity were simultaneously assessed at rest and during maximal hyperemia (ic. bolus of adenosine 40–60 μg) in patients with severe AS and unobstructed coronary arteries before and immediately after TAVR	27 symptomatic patients with severe AS and unobstructed CAD were included and compared with 28 patients without AS and unobstructed CAD (control group)	TAVR induces an immediate decrease in hyperemic microcirculatory resistance and an immediate increase in hyperemic flow velocity, whereas resting hemodynamics remain unaltered
Pesarini et al. (116)	Circ. Cardiovasc. Interv. 2016.	Prospective, observational study: the functional relevance of coronary lesions was simultaneously assessed by FFR using ic. bolus of adenosine 150–250 μg in patients with severe AS before and immediately after TAVR	54 symptomatic patients with severe AS and obstructive CAD were included	Post-TAVR functional assessment with conventional FFR cut-off may change the indication to perform PCI in around 15% of patients with CAD undergoing TAVR. Therefore, functional assessment with FFR may be more reliable after TAVR
Scarsini et al. (117)	Int. J. Cardiol. 2017	Prospective, observational study: the study aimed to compare the diagnostic performance of FFR and iFR in patients with severe AS and obstructive CAD. The iFR-FFR diagnostic agreement has been tested using the conventional FFR cut-off 0.80	85 patients with severe AS and 179 coronary lesions were included and compared with a control group formed by 167 patients (290 lesions) with stable CAD and without AS	The conventional iFR cut-off has lower diagnostic accuracy in the group of AS patients for detecting coronary lesion with FFR ≤ 0.80 in the group of CAD patients. The best diagnostic iFR cut-off was lower in the group of AS patients compared with the cut-off point observed in CAD patients (0.83 vs 0.89)
Scarsini et al. (118)	EuroIntervention 2018	Prospective, observational study: iFR and FFR using ic. bolus of adenosine 150–250 μg were measured in patients with severe AS and CAD before and immediately after TAVR	66 patients with severe AS and 145 coronary lesions were included	Higher iFR variation occurred mostly in patients with more severe aortic valve gradient and higher post-TAVR transaortic gradient drop. The iFR-FFR classification agreement is generally poorer in coronary stenosis with more severe angiographic and functional characteristics
Scarsini et al. (119)	Cardiovasc. Revasc. Med. 2018	Prospective, observational study: iFR and FFR using ic. bolus of adenosine 150–250 μg were measured in patients with severe AS and CAD before and immediately after TAVR. All decisions about revascularization were based on post-TAVR FFR assessment with a conventional cut-off 0.80.	62 patients with severe AS and concomitant CAD were included	A “defer iFR value” >0.93 yielded a NPV of 98% to exclude FFR non-significant stenosis (>0.80), and a “treatment iFR value” <0.83 had a PPV of 91% to identify FFR-significant stenosis (≤ 0.80). This hybrid decision-making strategy spared 63% of patients from adenosine, while maintaining 97% overall agreement with FFR lesions classification
Scarsini et al. (120)	Int. J. Cardiol. 2019	Prospective, observational study: FFR using iv. infusion of adenosine 140 μg/kg/min, iFR and adenosine-stress myocardial perfusion on SPECT were performed in patients with severe AS and borderline coronary lesions before TAVR	28 patients with severe AS and 41 borderline coronary lesions were included	FFR with conventional cut-off 0.80 was a better predictor of myocardial ischemia on SPECT (PPV 73%, NPV 95%) in comparison to iFR with conventional cut-off 0.89 (PPV 47%, NPV 91%). Using a lower iFR cut-off of 0.82 significantly improved its categorial agreement with the presence of myocardial ischemia on SPECT (from 59 to 73%) with an insignificant loss of its NPV (from 91 to 86%)
Ahmad et al. (105)	J. Am. Coll. Cardiol. Intv. 2018	Prospective, observational study: iFR, FFR, whole-cycle flow, systolic flow, wave-free period flow, microcirculatory resistance, at rest and during maximal hyperemia (ic. bolus of adenosine 150 μg) in patients with severe AS and CAD before and immediately after TAVR	28 patients with severe AS and 41 coronary lesions were included	Systolic and hyperemic coronary flow velocity increased significantly immediately after TAVR. Thus, hyperemic physiological indices that include systole underestimated coronary stenosis severity in patients with severe AS. After TAVR, iFR values remain unchanged, whereas FFR decreases significantly
Yamanaka et al. (121)	J. Am. Coll. Cardiol. Intv. 2018	Prospective, observational study: the study aimed to assess the diagnostic performance of iFR with adenosine-stress myocardial perfusion on SPECT and FFR cut-off ≤ 0.80 using iv. infusion of adenosine 140 μg/kg/min, in patients with severe AS and CAD	95 patients with severe AS and 116 intermediates coronary stenoses were included	iFR with a lower cut-off 0.82 could be a reliable diagnostic tool for indicating reversible myocardial perfusion defects on SPECT as well as FFR ≤ 0.80, in patients with severe AS
Vendrik et al. (103)	J Am. Heart. Assoc. 2020	Prospective, observational study: iFR FFR, whole-cycle flow, systolic flow, wave-free period flow, microcirculatory resistance, at rest and during maximal hyperemia (ic. bolus of adenosine 100–200 μg) in patients with severe AS and CAD before TAVR, immediately after TAVR and 6-months after TAVR	13 patients with severe AS and moderate-severe coronary lesions were included	Hyperemic coronary flow velocity increases immediately after TAVR and continues to rise to 6-month follow-up. This rise in flow causes both acute and long-term declines in FFR values, leading FFR to underestimate coronary stenosis severity in the presence of severe AS. Resting diastolic flow and consequently iFR are is not affected by severe AS and remain unchanged pre-TAVR, post-TAVR, and at 6-month follow-up.

*NPV, negative predictive value; PPV, positive predictive value; PCI, percutaneous coronary intervention; SPECT, single-photon emission computed tomography*.

Both FFR and iFR are pressure-derived indices which means that their measurements are based on a linear relationship between pressure and coronary flow under conditions of stable, constant and minimized intracoronary resistance (109, 110). Fractional flow reserve, estimated as the ratio of mean distal intracoronary to mean aortic pressure during hyperemia, is a hyperemic index measured over the whole cardiac cycle and includes systolic coronary flow (109). The use of adenosine as the most potent vasodilator of the intramyocardial arterioles, either as an intracoronary bolus at a dose of 150 to 250 μg or intravenous infusion at a dose of 140 μg/kg/min for at least 1 min, is safe and well-tolerated regarding adverse side effects in AS-patients with co-existing CAD (77, 103, 105, 116–124). Of note, a mild decrease in microcirculatory resistance and a moderate increase in coronary blood flow were documented during adenosine-induced maximal hyperemia in the coronary artery segment distal to the intermediate stenosis immediately after TAVR compared with pre-TAVR conditions (77, 105). Contrary, these coronary hemodynamics at rest remain unaltered following TAVR due to compensatory vasodilation of intramyocardial arterioles as a response to longstanding LV hypertrophy and subsequent capillary rarefaction (77, 105). Hence, the coronary vasodilatory capacity remains impaired after TAVR compared with normal subjects, but may be fully regained in the coming months or years with accompanying LV hypertrophy regression (77, 105). Moreover, the moderate improvement in hyperemic coronary blood flow after TAVR is mainly driven by an increase in hyperemic systolic coronary blood flow, which leads to a higher hyperemic whole-cycle flow and therefore lower FFR values compared with the pre-TAVR values (77, 105). As a result, any physiological index that includes the systolic phase of cardiac cycle will be affected by TAVR (105). These findings suggest the following mechanisms that may contribute to the higher FFR values in severe AS-patients before TAVR: (1) the presence of low resting microcirculatory resistance as a response to LV hypertrophy and increased LV oxygen demand to maintain resting coronary blood flow which means that vasodilatory capacity of microcirculation is already exhausted by the autoregulation phenomenon; (2) the presence of high hyperemic microcirculatory resistance due to structural and functional changes of the microcirculation (arteriolar remodeling, perivascular fibrosis, and capillary rarefaction); and (3) the high levels of circulating vasoconstrictors due to hyperactivation of sympathetic adrenergic and renin-angiotensin-aldosterone systems to increase vascular tone and maintain systemic arterial blood pressure, which may block or attenuate the vasodilatory effect of adenosine on microcirculation (67–69, 76, 77, 80, 105). Accordingly, the effect of adenosine is attenuated in the presence of AS, and therefore the blunted FFR before TAVR may underestimate coronary stenosis severity in patients with AS (67–69, 76, 77, 80, 105). Similarly, Pesarini et al. found that the mean FFR value was significantly lower after TAVR in patients with severe AS and co-existing intermediate-to-severe coronary stenosis defined as >50% DS assessed by quantitative coronary analysis (0.84 ± 0.12 vs. 0.82 ± 0.16; *p* = 0.02) (116). In contrast, mean FFR value remained unchanged after TAVR in AS-patients with angiographically non-significant coronary stenosis (<50% DS) (0.90 ± 0.07 vs. 0.91 ± 0.09; *p* = 0.69) (116). In the group of AS-patients and coronary stenosis with positive FFR values (below ischemic threshold ≤ 0.80) before TAVR, FFR was found to further deteriorate immediately after TAVR (0.71 ± 0.11 vs. 0.66 ± 0.14), whereas in those with negative FFR values (>0.80) before TAVR, it was slightly improved (0.92 ± 0.06 vs. 0.93 ± 0.07) (116). These variations in FFR values after TAVR crossed the threshold of 0.80 and changed the revascularization strategy in only 6% of patients with AS and coronary stenosis (116). Accordingly, the study suggests that FFR measured immediately after TAVR could be more suitable for the functional evaluation of coronary stenosis severity in these patients compared with FFR obtained in pre-TAVR clinical settings (116). However, it is questionable whether FFR may be reliably index for the functional assessment of coronary stenosis several days or months after TAVR because it has been shown that complete restoration of coronary vasodilatory capacity could be achieved only with longer-term post-TAVR following LV hypertrophy regression (70, 77, 98–102). Vendrik et al. also found an ongoing decrease in FFR immediately after TAVR and 6 months after the procedure compared with pre-TAVR values, whereas iFR and resting Pd/Pa remain unchanged (103). These findings suggest that FFR is a less reliable physiological index for the assessment of coronary lesion severity in patients with severe AS for at least 6 months after AVR (70, 77, 98–103). However, it remains unknown whether FFR could be suitable for the functional assessment of coronary lesions beyond 6 months after AVR.

**Figure 1:**
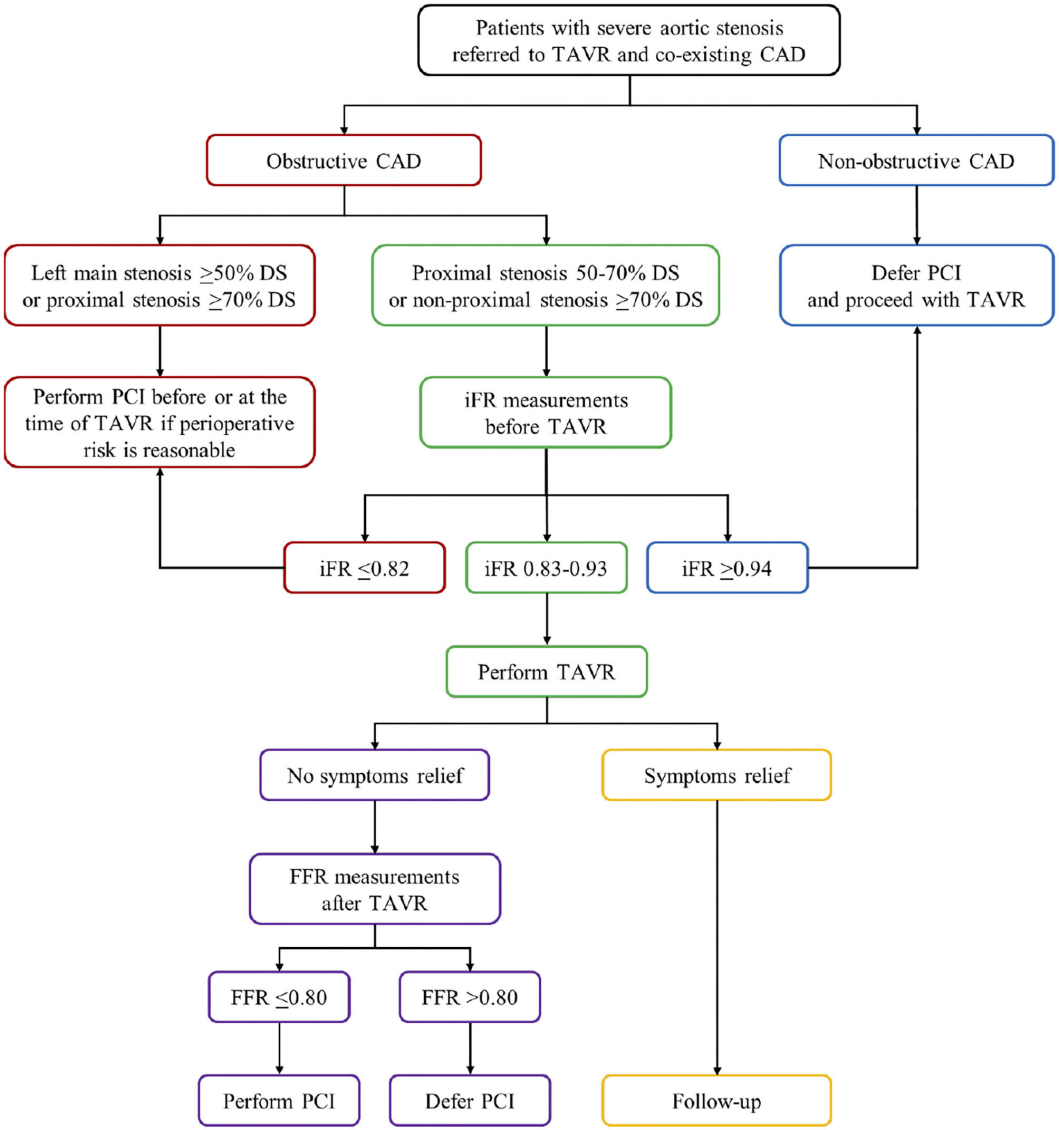
The proposed flow chart illustrates the myocardial revascularization strategy in patients with severe aortic stenosis undergoing TAVR. Obstructive CAD is defined as coronary artery stenosis ≥50% DS. CAD, coronary artery disease; DS, diameter stenosis; FFR, fractional flow reserve; iFR, instantaneous wave-free ratio; PCI, percutaneous coronary intervention; TAVR, transcatheter aortic valve replacement.

Unlike FFR, resting coronary hemodynamics including resting coronary flow and resting microcirculatory resistance during the whole diastole as well as during the wave-free period of diastole are not significantly affected by the presence of severe AS and remain unaltered before TAVR, immediately after TAVR, and at 6 months follow-up (77, 96, 97, 103, 105). Accordingly, it has been shown that iFR as a non-hyperemic index is independent of both AS severity and TAVR in short- and long-term follow-up (103, 105). The iFR is defined as the ratio of mean distal intracoronary to mean aortic pressure measured under resting conditions during a specific wave-free period of diastole when microcirculatory resistance is stable and minimized (110). Coronary blood flow during this period occurs when the aortic valve is closed while the myocardium is completely relaxed and without contraction (103). Therefore, it is reasonable to believe that iFR is a more reliable physiological index for the assessment of coronary stenosis severity in the presence of AS (77, 96, 97, 103, 105). Scarsini et al. also found that mean iFR values did not change before and after TAVR, although individual iFR measurements showed high and inconsistent variations following TAVR in around 15% of coronary lesions, mainly in AS-patients with angiographically intermediate severity (37–70% DS) (118). Both negative (iFR >0.89) and positive iFR values (iFR ≤ 0.89) before TAVR crossed below or above the ischemic threshold 0.89 after TAVR in similar percent of coronary lesions (6.9% vs. 7.3%, respectively) and, thereby, changed the revascularization strategy (118). These high iFR variations occurred mostly in patients with more severe aortic valve gradients and higher post-TAVR transaortic gradient drops reminding that iFR measurements must be carefully taken in this subgroup of AS-patients (118). The same study also showed that iFR with a conventional cut-off of 0.89 had a high NPV in both pre-TAVR and post-TAVR settings (99% vs. 97%) for excluding without risk the presence of functionally significant coronary lesions defined as FFR ≤ 0.80 (118). However, the low PPV of iFR for the detection of significant coronary lesions in AS-patients in both settings (44% vs. 60%, respectively) indicates that predefined ischemic threshold of 0.89 for the assessment of FFR-defined lesion severity may not be appropriate (27, 118). The same authors also presented a high NPV and a low PPV of iFR with a conventional cut-off of 0.89 (91% and 47%) for identifying myocardial ischemia on SPECT in the presence of AS (120). Discordance between iFR and SPECT was found in 41% of patients and 95% of them had false-positive iFR values (negative SPECT and iFR ≤ 0.89) (120). The higher rate of false-positive iFR values in severe AS-patients could be explained by increased resting coronary blood flow in response to the increased LV oxygen demand due to higher LV afterload and LV hypertrophy (77, 99, 120). Consequently, a higher pressure gradient occurs across the coronary lesion leading to a lower CFR as well as iFR (120). Hence, the iFR may overestimate coronary stenosis severity in patients with severe AS. To overcome these limitations of iFR, several authors proposed a lower ischemic threshold to achieve a higher positive predictive value in the presence of AS (27, 118). The other study conducted by the same authors revealed that shifting the iFR cut-off from 0.89 to 0.83 in patients with severe AS and co-existing CAD significantly increase its categorial agreement with FFR using cut-off ≤ 0.80 measured in patients with CAD but without AS (control group), from 76% to 91%, while maintaining its NPV (95%) (117). Yamanaka et al. evaluated the diagnostic performance of iFR in 95 patients with severe AS and concomitant intermediate coronary lesions, as compared with myocardial perfusion scintigraphy and with FFR as reference (121). They demonstrated that the optimal cut-off value of iFR for detecting the presence of myocardial ischemia on myocardial perfusion scintigraphy was 0.82 (AUC: 0.84). Similarly, the same iFR cut-off was optimal for indicating an FFR ≤ 0.75 and ≤ 0.80 with an AUC of 0.92 and 0.82, respectively (121). The study concluded that iFR with a lower ischemic threshold of 0.82 has excellent reproducibility and could be used as a reliable physiological index for the assessment of coronary lesion severity in the presence of severe AS (121). Scarsini et al. also noted that using a lower iFR ischemic threshold of 0.82 in these patients significantly improved its categorial agreement with the presence of myocardial ischemia on SPECT (from 59% to 73%) with an insignificant loss of its NPV (from 91% to 86%) (120). However, they regained the use of FFR with a lower cut-off 0.78 as a more accurate physiological index for detecting myocardial ischemia in patients with severe AS and CAD compared with iFR using cut-off 0.82 (AUC: 88% vs. 73%; NPV: 92% vs. 86%; PPV: 81% vs. 73%) (120). This study is hampered by the fact that FFR has not been so far validated in the presence of AS and the conventional or lower FFR threshold (0.80 vs. 0.78) might not accurately reflect the coronary stenosis severity (120). The same authors proposed a new iFR-FFR “hybrid approach” with the iFR measurements before TAVR as the first choice for the functional assessment of coronary stenosis in the presence of severe AS (119). They found that the iFR threshold >0.93 had an NPV of 98% to exclude significant stenosis defined as post-TAVR FFR ≤ 0.80. Contrary, iFR threshold <0.83 had a PPV of 91% to identify FFR-defined significant stenosis after TAVR (119). Accordingly, FFR was used only when iFR values were between 0.83 and 0.93 (119). This “hybrid” approach enables the assessment of coronary stenosis severity without vasodilatory provocation in 63% of patients with severe AS while maintaining 97% overall agreement with FFR lesions classification (Figure 1) (119).

In summary, to determine the optimal FFR and iFR ischemic thresholds in patients with severe AS and co-existing CAD, additional prospective randomized trials are needed with a larger number of patients. Both physiological indices must be validated with cardiovascular events during long-term follow-up by randomized trials comparing FFR-guided and/or iFR-guided myocardial revascularization with angiographically-guided therapy in patients with severe AS [FAITAVI (Functional Assessment in TAVI; NCT03360591) trial]. Furthermore, the prognostic relevance of PCI before or after TAVR remains controversial, and several clinical trials regarding optimal time for PCI in patients referred to TAVR are still ongoing [the NOTION-3 (Nordic Aortic Valve Intervention-3; NCT03058627) trial; the REVIVAL (Revascularization After Transcatheter Aortic Valve Implantation; NCT03283501) trial; the TCW (The TransCatheter Valve and Vessels Trial; NCT03424941) trial; the TAVI-PCI (Optimal Timing of Transcatheter Aortic Valve Implantation and Percutaneous Coronary Intervention; NCT04310046) trial].

## Author Contributions

SA and MB: conceptualization. SA: resources and writing—original draft preparation. MB and BB: writing—review and editing. BB: supervision. All authors participated in writing and critically reviewing this manuscript. All authors have read and agreed to the published version of the manuscript.

## Conflict of Interest

The authors declare that the research was conducted in the absence of any commercial or financial relationships that could be construed as a potential conflict of interest.

## Publisher's Note

All claims expressed in this article are solely those of the authors and do not necessarily represent those of their affiliated organizations, or those of the publisher, the editors and the reviewers. Any product that may be evaluated in this article, or claim that may be made by its manufacturer, is not guaranteed or endorsed by the publisher.
